# A homologous mapping method for three-dimensional reconstruction of protein networks reveals disease-associated mutations

**DOI:** 10.1186/s12918-018-0537-2

**Published:** 2018-03-19

**Authors:** Sing-Han Huang, Yu-Shu Lo, Yong-Chun Luo, Yu-Yao Tseng, Jinn-Moon Yang

**Affiliations:** 1 Institute of Bioinformatics and Systems Biology, National Chiao Tung University, Hsinchu, 30050 Taiwan; 2 Department of Biological Science and Technology, National Chiao Tung University, Hsinchu, 30050 Taiwan

**Keywords:** Structural systems biology, Structurally resolved PPI networks, Homologous mapping method, Disease-associated proteins with mutations

## Abstract

**Background:**

One of the crucial steps toward understanding the associations among molecular interactions, pathways, and diseases in a cell is to investigate detailed atomic protein-protein interactions (PPIs) in the structural interactome. Despite the availability of large-scale methods for analyzing PPI networks, these methods often focused on PPI networks using genome-scale data and/or known experimental PPIs. However, these methods are unable to provide structurally resolved interaction residues and their conservations in PPI networks.

**Results:**

Here, we reconstructed a human three-dimensional (3D) structural PPI network (hDiSNet) with the detailed atomic binding models and disease-associated mutations by enhancing our PPI families and 3D–domain interologs from 60,618 structural complexes and complete genome database with 6,352,363 protein sequences across 2274 species. hDiSNet is a scale-free network (γ = 2.05), which consists of 5177 proteins and 19,239 PPIs with 5843 mutations. These 19,239 structurally resolved PPIs not only expanded the number of PPIs compared to present structural PPI network, but also achieved higher agreement with gene ontology similarities and higher co-expression correlation than the ones of 181,868 experimental PPIs recorded in public databases. Among 5843 mutations, 1653 and 790 mutations involved in interacting domains and contacting residues, respectively, are highly related to diseases. Our hDiSNet can provide detailed atomic interactions of human disease and their associated proteins with mutations. Our results show that the disease-related mutations are often located at the contacting residues forming the hydrogen bonds or conserved in the PPI family. In addition, hDiSNet provides the insights of the FGFR (EGFR)-MAPK pathway for interpreting the mechanisms of breast cancer and ErbB signaling pathway in brain cancer.

**Conclusions:**

Our results demonstrate that hDiSNet can explore structural-based interactions insights for understanding the mechanisms of disease-associated proteins and their mutations. We believe that our method is useful to reconstruct structurally resolved PPI networks for interpreting structural genomics and disease associations.

**Electronic supplementary material:**

The online version of this article (10.1186/s12918-018-0537-2) contains supplementary material, which is available to authorized users.

## Background

One of the crucial steps toward understanding the associations among molecular interactions, pathways, and diseases in a cell is to investigate detailed atomic protein-protein interactions (PPI) in the structural interactome. Many high-throughput experimental methods, such as high-throughput yeast two-hybrid screening [1, 2] and co-affinity purification [3], and computational approaches have been proposed to generate large-scale PPIs. The PPIs identified from these experimental methods are often unable to reflect the binding mechanisms of PPI that how a protein interacts with another one, and could not describe the relationship between mutated protein and disease.

**Fig. 1:**
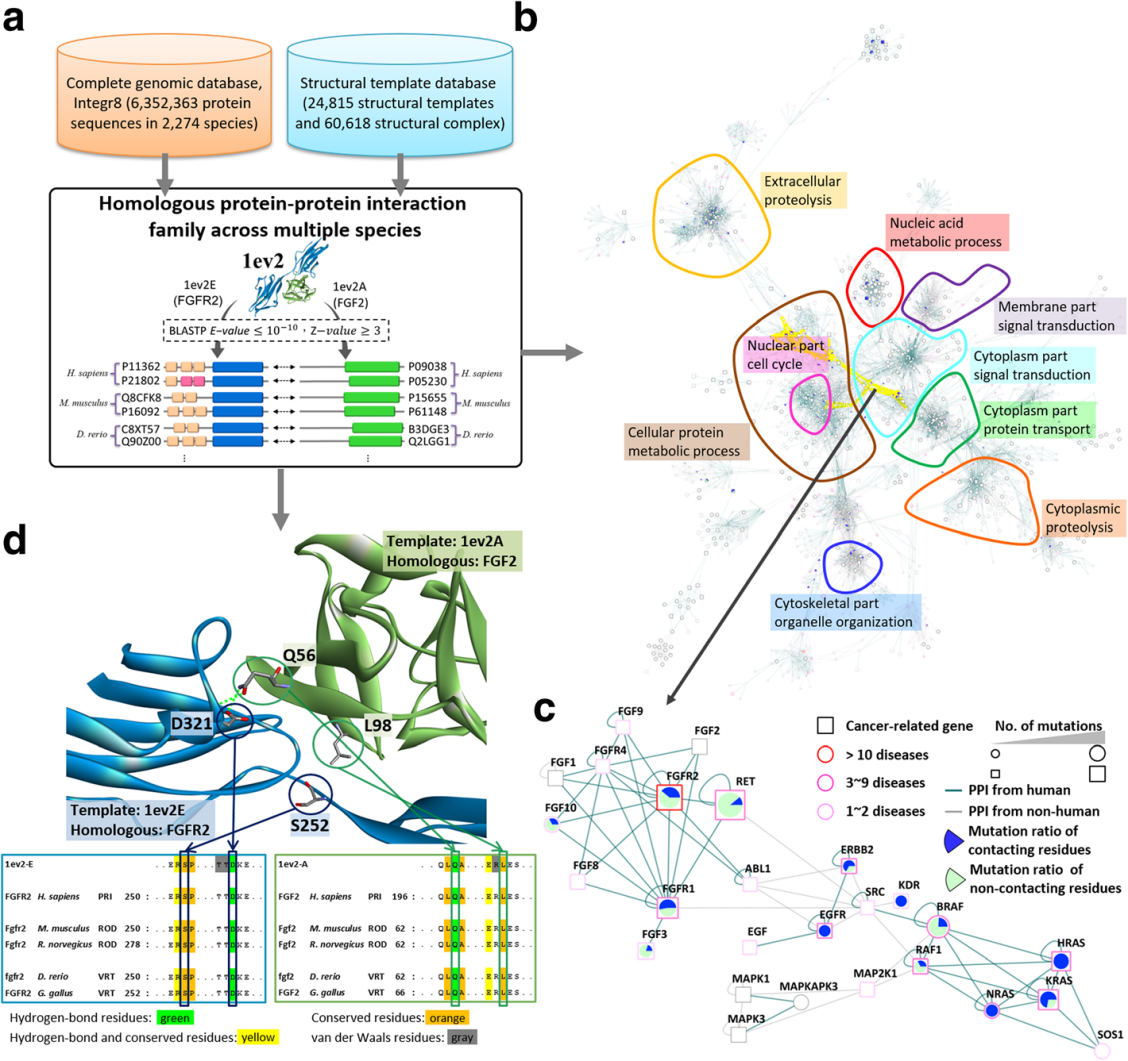
Overview of reconstructing human structural interactome (hDiSNet) using 3D–domain interolog mapping. **a** 3D–domain interolog mapping infers human PPIs through 60,618 three-dimensional (3D) structural complexes and complete genome database with 6,352,363 protein sequences across 2274 species using the 3D–template 1ev2 complex as the example. We totally infer the 19,239 PPIs in 5177 proteins in the reconstructed human structural interactome (hDiSNet) from 60,618 structural templates. **b** The largest sub-network of hDiSNet with 2051 proteins and 11,534 PPIs can be grouped into nine major cellular processes, including extracellular proteolysis (yellow), nucleic acid metabolic (red), cellular protein metabolic (brown), nuclear part cell cycle (pink), membrane signal transduction (purple), cytoplasm part signal transduction (cyan), cytoplasm part signal protein transport (green), cytoplasmic proteolysis (orange), and cytoskeletal part organelle organization (blue). **c** The FGFR (EGFR)-MAPK pathway in hDiSNet. The node sizes of circle (proteins) and box (cancer-related proteins) denote the numbers of mutations recorded in OMIM database. The colored borders of nodes indicate the numbers of diseases associated proteins. The colored nodes indicate the ratios of mutations in the contacting and non-contacting residues. **d** Detailed atomic interactions of FGFR2-FGF2 and the MSA of contacting residues across multiple species. The contacting residues are colored according to their types: for forming hydrogen bonds (green), conserved (orange), both for forming hydrogen bonds and conserved (yellow), and for forming van der Waals force (gray)

**Fig. 2:**
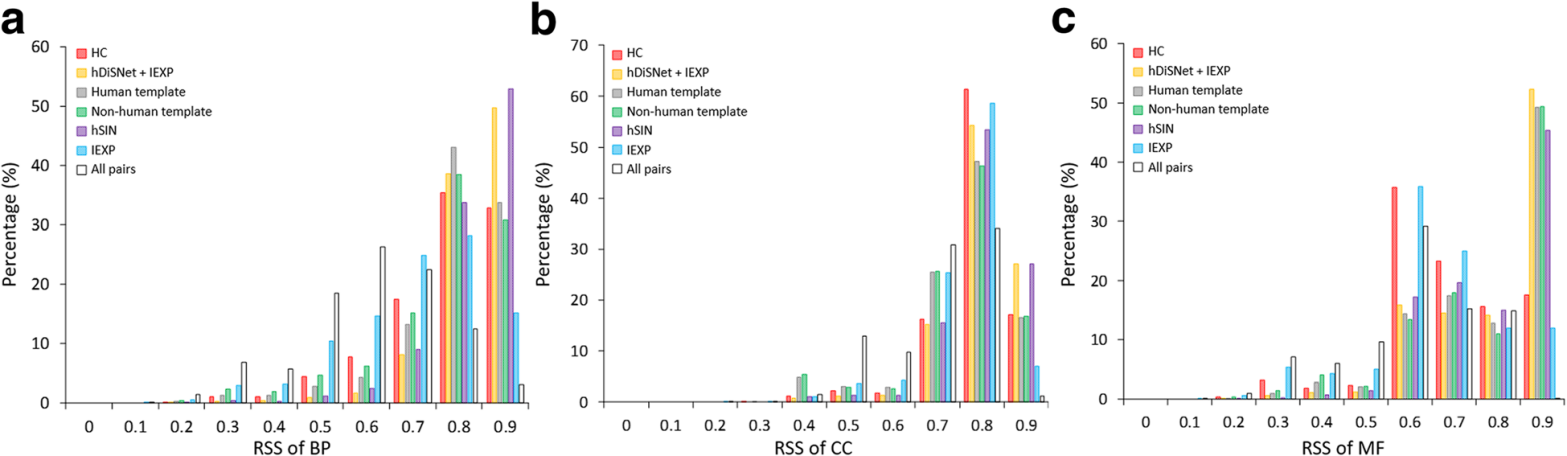
Comparisons of our predicted PPIs with reference PPI sets. The relative specificity similarity (RSS) shows the distributions of (**a**) biological process (BP), (**b**) cellular component (CC), and (**c**) molecular function (MF) of our hDiSNet and reference PPI sets, including our predicted PPIs using human (10,651 PPIs, gray) and non-human (8588 PPIs, green) templates, 4375 overlapped PPIs between hDiSNet and IEXP PPIs (yellow), 25,675 HC (red) PPIs, 181,868 IEXP (blue) PPIs, 4222 hSIN (purple) PPIs, and 2,102,275 all possible PPI pairs (white)

**Fig. 3:**
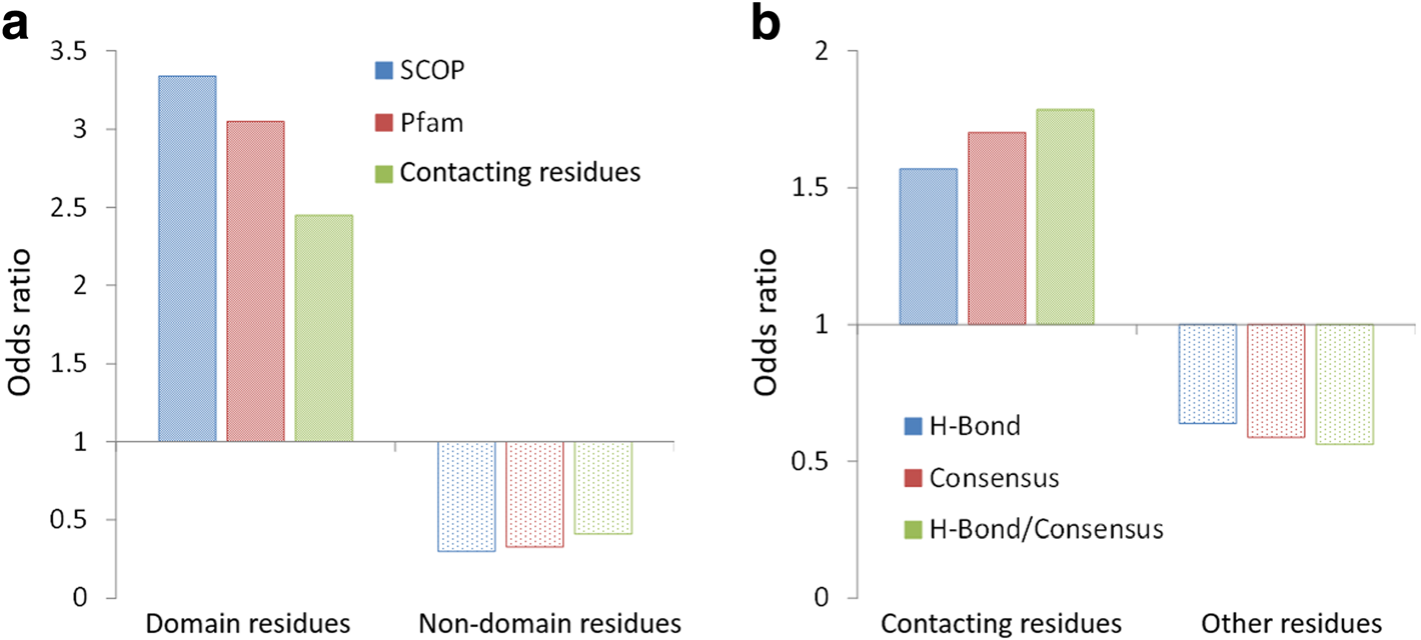
The enrichment (odds ratio) of our predicted interacting domains and contacting residues by using 5843 mutations recorded in OMIM. **a** The odds ratios of in-frame mutations in interacting domains (SCOP with blue and Pfam with red), contacting residues (green), non-interacting domains (SCOP with dot blue and Pfam with dot red), and non-contacting residues (dot green). **b** The odds ratios of in-frame mutations are colored according to their types: contacting residues for forming hydrogen-bond (blue), conserved (red), and for forming hydrogen-bond and conserved (green); other residues for forming hydrogen-bond (dot blue), conserved (dot red), and for forming hydrogen-bond and conserved (dot green). The odds ratios of in-frame mutations are significantly enriched in the interacting domains and contacting residues

**Fig. 4:**
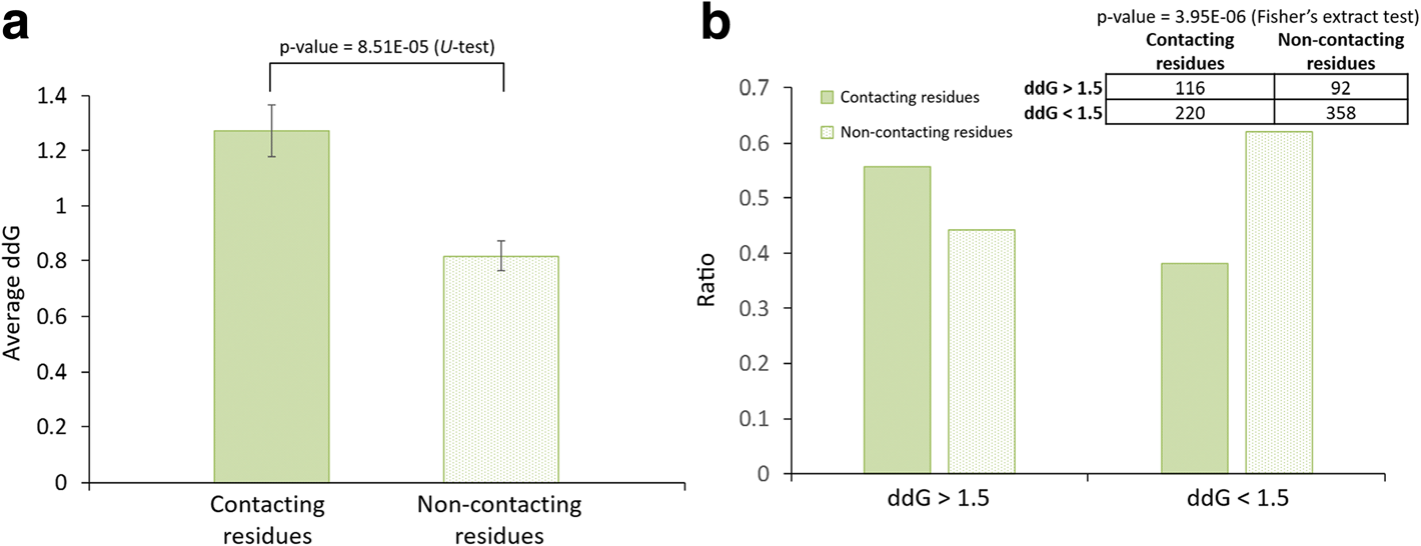
Our identified contacting and non-contacting residues association with binding affinity. **a** The average ddGs of contacting and non-contacting residues are 1.27 and 0.82 (*p*-value = 8.51E-05 by U-test), respectively. **b** The ratios of contacting and non-contacting residues are 0.56 (116/208) and 0.44 (92/208) in ddG > 1.5, respectively, and 0.38 (220/578) and 0.62 (358/578) in ddG < 1.5. The contacting residues are significantly enriched in ddG > 1.5 (*p*-value = 3.95E-06 by Fisher’s exact test)

**Fig. 5:**
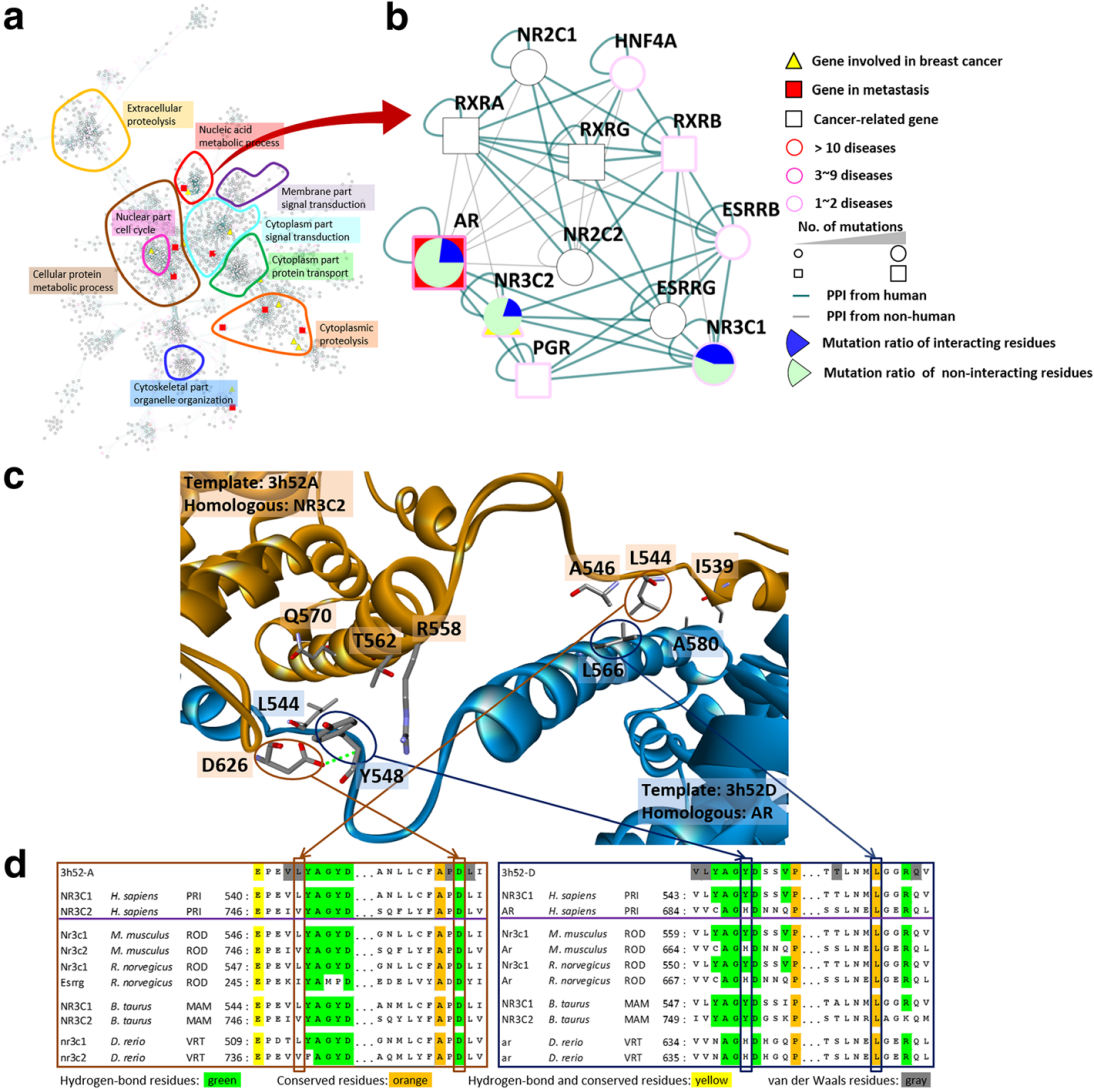
Three-dimensional reconstruction of PPI network for breast cancer. **a** The breast cancer (red boxes) and metastasis (yellow triangles) related proteins are located in five regions, including cytoplasm part signal transduction (cyan), cytoplasmic proteolysis (orange), cellular protein metabolic process (brown), nucleic acid metabolic process (red), and nuclear part cell cycle (pink). **b** The AR-regulated sub-network contains androgen receptor (AR), Nuclear receptor subfamily 3 group C member 2 (NR3C2), and their interacting proteins in the nucleic acid metabolic process. The mutations of seven proteins (e.g. AR, RXRA and PGR) in AR-regulated sub-network are considered as disease-association (e.g. breast cancer, glucocorticoid resistance, and progesterone resistance) in OMIM. **c** Detailed atomic interactions of AR-NR3C2 and the contacting residues analysis using the 3D–template complex with steroid-binding domain (PDB code: 3 h52). **d** The MSA of PPI family of proteins NR3C2 and AR. The contacting residues are colored according to their types: for forming hydrogen bonds (green), conserved (orange), for forming van der Waals force (gray), and for forming hydrogen bonds and conserved (yellow)

As the increase in the number of available three-dimensional (3D) structural complexes, there is a new opportunity to develop a fast and accurate computational method for inferring structurally resolved PPIs and constructing structural PPI networks. The structural complexes provide domain-domain interactions and atomic details for thousands of direct physical PPI interactions. Several works have combined protein structures with experimental PPIs to study how mutations affect protein interactions in diseases [4, 5]. For example, Wang et al. considered both structural complexes with known interacting domains (e.g., iPfam) and high-quality binary interactions, from literature and yeast two-hybrid screens, to construct human structural interaction network (hSIN), and they mapped disease-related mutations into the proposed network [4]. To study mutated proteins in PPI networks, the human structural interactome provides detailed atomic interactions to examine the linkage between the disease-related mutations and protein binding mechanisms. Some methods have utilized template-based methods to predict the PPIs by accessing interface preference through the fitness of known template structures [6]. However, these methods are time-consuming to search for all possible protein-protein pairs in a large genome-scale database to construct the human structural interactome. Recently, we have proposed 3D–domain interologs with the template-based scoring function to infer the binding models of homologous PPI (called PPI family) of a 3D complex structure by comparative modeling across multiple species [7].

Here, we propose a structural systems biology method for reconstructing human structural interactome (hDiSNet) with physical PPIs by enhancing our previous 3D–domain interologs and scoring functions [7]. We collected a structural template library comprising 60,618 3D–dimers from the protein data bank (PDB) and the complete genomic database (Integr8 [8], with 6,352,363 protein sequences in 2274 species). hDiSNet consists of 5177 proteins and 19,239 predicted PPIs with 5843 mutations recorded in the Online Mendelian Inheritance in Man (OMIM) [9] and 42,688 mutations in the Catalogue Of Somatic Mutations In Cancer (COSMIC) [10]. These predicted 19,239 PPIs share the higher Gene Ontology similarities and co-expression correlations than the ones of using 181,868 experimental PPIs recorded in five public databases (i.e., IntAct, MIPS, DIP, MINT, and BioGRID) [11–15]. In addition, our hDiSNet enlarge the number of PPIs more than 4 times compared to present structural PPI network (i.e. hSIN [4], 4222 PPIs). Moreover, our proposed network is a scale-free network (γ = 2.05), which is consistent with the architecture of cellular networks. Among 5843 mutations from OMIM, 1653 and 790 mutations involved in interacting domains and contacting residues, respectively, are highly related to diseases. According to 42,688 cancer-related somatic mutations derived from COSMIC, we found that 14,684 and 5883 mutations are located at interacting domains and contacting residues, respectively. Furthermore, the disease-related mutations are more enriched in the residues that are able to form the hydrogen bonds and are conserved across multiple organisms. Our structurally resolved PPI network (hDiSNet) provide the insights for interpreting the mechanisms of breast cancer and ErbB signaling pathway for brain cancer. These results indicate that our method is useful to reconstruct structural PPI network for understanding the associations between mutations and diseases. The reconstructed human structural interactome (hDiSNet) and other supporting data are available at http://gemdock.life.nctu.edu.tw/3d-network.

## Methods

### Overview

Figure 1 illustrates the overview of reconstructing human structural interactome (hDiSNet) though “3D–domain interolog mapping”. First, a structural template library comprising 60,618 3D–dimers involved in 24,815 complexes was selected from PDB released on Sep 2, 2011 (Fig. 1a). For a given 3D–dimer (e.g., FGFR2-FGF2, PDB code: 1ev2), we identified the homologous proteins (i.e., BLAST *E*-value < 10^− 10^) from the Integr8 complete genomic database [8], including 6,352,363 protein sequences in 2274 species, and our scoring functions [7, 16] were used to infer the contacting residues and evaluate the similarities of binding interfaces (i.e., *Z*-value ≥3.0). According to these homologous PPIs, we reconstruct the human structural interactome (hDiSNet). This network consists of 5177 proteins and 19,239 PPIs and the largest sub-network includes 2051 proteins and 11,534 PPIs (Fig. 1b). The node (protein) in the network indicates the ratio of the mutations on the contacting and non-contacting residues and the number of mutations and diseases. The edges (i.e. PPIs) indicate the PPIs inferred from the human and non-human templates. The maximum sub-network can be grouped into nine major cellular processes, including extracellular proteolysis (yellow), nucleic acid metabolic (red), cellular protein metabolic (brown), nuclear part cell cycle (pink), membrane signal transduction (purple), cytoplasm part signal transduction (cyan), cytoplasm part signal protein transport (green), cytoplasmic proteolysis (orange), and cytoskeletal part organelle organization (blue). Based on mutations (i.e., SNPs) and the reconstructed human structural PPI network, we inferred the mutations-disease associations with detailed atomic binding models. Our human structural PPI network reflects the mutation-disease associations on EGFR (FGFR)-MAPK pathway (Fig. 1c). Among 26 proteins in EGFR (FGFR)-MAPK pathway, 21 proteins (e.g., FGFR1, FGFR2, EGFR, and RET) have been identified as the cancer-related proteins [17] and 10 proteins (e.g. FGFR1, FGFR2, EGFR, ERBB2) have more than 3 disease-related mutations (Additional file 1: Table S1). Because the mutations in interacting domains or interaction sites often disrupt the PPI, the reconstructed human structural PPI network could interpret the mechanisms of disease-associated proteins and their mutations (Fig. 1d).

### 3D–domain interolog mapping and scoring function

To efficiently enlarge protein interactions annotated with residue-based binding models, we have previously proposed the concept “3D–domain interolog mapping” [7, 16]: for a known 3D–structure complex (template T with chains A and B), domain *a* (in chain A) interacts with domain *b* (in chain B) across multiple species. The proteins of the homolog families A’ and B′ of A and B have the significant sequence similarity (i.e. BLASTP *E*-values ≤10^− 10^) and contain interacting domains *a* and *b*, respectively. All possible protein pairs between these two homolog families are considered as protein-protein interaction candidates using the template T. Then, we utilize our previous scoring system [7, 16] to evaluate the binding model similarity between candidates and template. The scoring function is briefly described as follows: *E*_*tot*_ = *E*_*vdw*_ + *E*_*SF*_ + *E*_*sim*_ + *E*_*cons*_, where *E*_*vdw*_ and *E*_*SF*_ are van der Waals and hydrogen-bond/electrostatic energies, respectively. The *E*_*sim*_ is the template interface similar score based on the aligned-contact residues of proteins A and B aligned to the hit template. The *E*_*cons*_ is couple-conserved residue score. The *E*_*vdw*_ and *E*_*SF*_ are residue-based energy functions, including sidechain-sidechain and sidechain-backbone energies [7, 16].

### Protein-protein interaction data sets

To investigate the reliability of PPIs derived from “3D–domain interolog mapping”, we compared these predicted PPIs with the experimental PPIs. In this paper, the 181,868 integrated experimental (IEXP) PPIs among 16,433 human proteins were collected from the five public databases (i.e., IntAct [11], MIPS [12], DIP [13], MINT [14], and BioGRID [15]; Additional file 1: Table S2). In addition, we compiled the high-confidence (HC) set, which was reported at least two different publications, including 25,675 high-confidence PPIs and 8965 proteins. Based on the human structural templates, we divided our predicted 19,239 PPIs into 10,651 PPIs from human-templates and 8588 PPIs from non-human-templates. To compare our hDiSNet with the present human structurally resolved PPI networks, we collected the hSIN, including 2816 proteins and 4222 PPIs, from Wang et al. [4].

### Protein-protein interactions in gene expression profiles

Moreover, to further assess the quality of our network, we collected three microarray sets, from GEO database (GSE12667 [18], GSE12276 [19], and GSE7696 [20]), which were conducted on various tissues to compare co-expression correlation of PPIs derived from PPI families and experimental data. We used these gene expressions to evaluate predicted PPIs derived from our methods. The co-expression value of PPIs is one of an index to measure the two proteins activated or non-activated simultaneously in specific states. For GSE12667 published in Nature journal by Li et al., the author used 188 lung cancer samples to identify the 26 potential genes in 188-paired tumor and normal subjects [18]. For GSE12276 published in Nature journal by Paula et al.**,** the author used 204 primary tumors from breast cancer patients to study the mechanism of breast cancer metastasis to the brain [19]. GSE7696 used 80 glioblastoma multiforme (GBM) samples to identify novel genes related to the malignant behavior of GBM [20]. In summary, two of the three datasets have high reliability based on high-impact journal, and all three datasets are comprised of a large number of samples, which ensure the robustness of our analysis.

### Disease-associated genes and mutations

To study the relationship between disease-associated proteins (genes) and their mutations in hDiSNet, we collected the disease-related mutations of these proteins from OMIM database [9]. The database of single nucleotide polymorphisms (dbSNP [21], build 132) is a public-domain archive for a broad collection of germline and somatic mutations associated with diseases. We collected 18,543 mutations including in-frame and truncating mutations in 2900 genes with “OMIM-curated-records” annotations from the dbSNP database. According to these 18,543 mutations, there are 5843 mutations associated with 776 genes in hDiSNet. To further examine the somatic mutation and cancer associations, we collected 91,000 somatic mutations (missense) with 546 cancer genes from the Cancer Gene Census (CGC) in the COSMIC database [10]. Here, total 42,688 mutations with 266 cancer genes were mapped to our hDiSNet. To investigate the enrichment of mutations in the interacting domains and contacting residues, we calculated the odds ratios of the mutations in the interacting domains, non-interacting domains, contacting residues, and non-contacting residues. Odds ratios were calculated by using the following equations:

1 $$ \mathrm{Odds}\kern0.17em \mathrm{ratio}=\frac{p_1/\left(1-{p}_1\right)}{p_2/\left(1-{p}_2\right)}, $$

where the *p*_*1*_ is the number of observed mutations in the interacting domains or contacting residues, and divided by the total number of mutations. The *p*_*2*_ is the total number of residues in the interacting domains or contacting residues, and divided by the length of all proteins combined. For example, the odds ratio of mutations in the interacting domain is calculated as follows: The value of *p*_*1*_ is 0.71 (1653/2330) of the Pfam domain residues (Additional file 1: Table S3). The value of *p*_*2*_ is 0.44 (125,606/282,517) of the Pfam domain residues. Therefore, the odds ratios of Pfam domain residue is 3.05 ([0.71/(1–0.71)]/[0.44/(1–0.44)]).

## Results and discussions

### hDiSNet: The reconstructed human structural interactome

The interacting proteins are usually involved in similar biological processes and located in similar cellular components. To verify the quality of our human structural interactome (hDiSNet), we calculated the relative specificity similarity (RSS) [22] of Gene Ontology (GO) [23], including biological process (BP), cellular component (CC), and molecular function (MF), in interacting protein pairs and all possible protein pairs. In addition, the 19,239 structurally resolved PPIs derived from our PPI families could be separated by the template organism of PPI family, which were 10,651 PPIs from human templates and 8588 from non-human templates. Figure 2 illustrates the RSS score distributions of BP, CC, and MF of our inferred PPIs derived from human and non-human templates, IEXP PPIs, HC PPIs, overlapped PPIs between our inferred and IEXP PPIs, hSIN, and all protein pairs. The distributions of RSS scores of PPIs derived from human (mean RSS scores = 0.83; gray) and non-human (0.81; green) templates are significantly more enriched than all protein pairs (0.62; white) in RSS-BP, CC, and MF (*p*-value < 0.01, Mann–Whitney *U* test). We also observed that PPIs derived from human and non-human templates are enriched than IEXP PPIs while the RSS-BP, CC, and MF are higher than 0.9 (Fig. 2). These results imply that the PPIs in our hDiSNet significantly share the similar biological functions than IEXP PPIs and all protein pairs. In addition, we found that the RSS-BP and RSS-MF scores of 4375 overlapped PPIs of hDiSNet and IEXP (yellow) are significantly different comparing to the scores of IEXP (*p*-value < 0.05) and all pairs (*p*-value < 0.01) (Fig. 2a and c). The RSS-BP, CC and MF scores of 4375 overlapped PPIs have no difference compared with that of 4222 PPIs from hSIN (*p*-value > 0.1; purple). In RSS-BP score > 0.9, the ratio of overlapped PPIs (0.50) are similar to hSIN (0.53), and more enriched than HC (0.33), IEXP (0.15) and all pairs (0.03). The results indicate that our reconstructed human structural interactome (hDiSNet) comprehensively includes 14,864 newly discovered PPIs and 4375 overlapped PPIs that enhance the Gene Ontology similarities of PPIs.

Furthermore, we have found that the reliability of a predicted PPI depends on the evolutionary distance between the target and source species based on our previous studies. Here, we compared the similarities of GO annotations of PPIs derived from human and non-human templates. The average of RSS-BPs of PPIs from human and non-human structural templates is 0.85 and 0.82, respectively. We found that the RSS-BPs of PPIs derived from human templates have no significant difference comparing with the non-human templates (Fig. 2a; *p*-value = 0.97). These results show that both of our predicted PPIs derived from human and non-human templates are robust and helpful for investigating the cellular processes.

To further assess the quality of our hDiSNet, we used three microarray sets, including lung, breast and brain cancer, for comparing co-expression correlation in our predicted PPIs and other PPI sets. (Additional file 1: Figure S1) illustrates the co-expression correlation of PPIs derived from human and non-human templates, hSIN, and experimental PPI sets (i.e., HC and IEXP). Our predicted PPIs derived from human (mean correlation = 0.06; gray) and non-human (mean correlation = 0.06; green) templates have no significant difference (*p*-value = 0.71) on co-expression of three different gene expression datasets. In addition, the HC PPIs have no difference (mean correlation = 0.07) compared with our inferred PPIs from PPI families (mean correlation = 0.06; *p*-value > 0.1), and our predicted PPIs have significant difference with the IEXP PPIs (mean correlation = 0.04; *p*-value < 0.05; blue) and significant higher correlation than all protein pairs (mean correlation = 0.01; *p*-value < 0.001; black dot lines). Furthermore, the co-expression correlation of 4375 overlapped PPIs from our inferred and IEXP (yellow) has no significant difference compared with that of hSIN (*p*-value > 0.5; purple), but has significantly higher correlation than that of IEXP (p-value < 0.01) and all protein pairs (*p*-value < 0.001). These results imply that both of our predicted PPIs derived from human and non-human templates are reliable and they could reflect the specific states of disease in time and space. Moreover, our hDiSNet completely comprises 14,864 newly discovered PPIs and 4375 overlapped PPIs that improve the co-expression correlation of PPIs.

A network with a power degree distribution is called scale-free, a name that is rooted in the statistical physics literature. An important finding of the cellular network architecture is that most networks within the cell approximate a scale-free topology [24]. Therefore, our hDiSNet was evaluated based on the characteristic of scale-free networks that the *P*(*k*), the probability of a node with *k* links, decreases as the node degree increases on a logarithmic scale plot (Additional file 1: Figure S2A)*.* The degree exponent γ is 2.05 in hDiSNet, as well as hSIN, HC, and IEXP are 2.23, 1.48, and 1.43, respectively (Additional file 1: Figures S2B-S2D). In addition, we found that our hDiSNet (19,239 PPIs) has expanded the number of PPIs more than 4 times compared to hSIN (4222 PPIs). These results show that hDiSNet and hSIN are satisfied with the properites of scale-free networks, which typically have degree exponents 2 ≤ γ ≤ 3, and are consistent with the architecture of some cellular networks [24, 25]. Furthermore, the results suggest that we not only make the present human structural PPI network more comprehensive, but we also keep the property of biological network and enrich the biological significance of the network.

### Mutation-disease associations on EGFR (FGFR)-MAPK pathway

Somatic mutations in proteins have been considered as one of the main causes for cancer development [26]. When a protein occurs mutation, it can influence linked PPIs and pathways to lead abnormal biological functions. According to previous studies, somatic mutations in a protein usually involved in some types (e.g., point mutation, deletion, and insertion) and occurred at different mutation sites (e.g., contacting residues and non-contacting residue). The mutations positioning on interacting domains or interaction sites often disrupt the protein-protein interactions and we considered these mutations as “hot spot of mutations”, which play potential roles to result in disease occurrence.

Based on our reconstructed human structural interactome (hDiSNet) with mutations and diseases, we identified two major regions which are signal receiving receptors (e.g., FGFR, EGFR, and RET) and their downstream signal transduction proteins (e.g., RAS, BRAF, and MAPK) (Fig. 1c). These two regions are linked with some PPIs (e.g., RET-SRC, EGFR-SRC, EGFR-ABL1) in which RET and EGFR are cancer-related proteins of “hot spot of mutations”. RET is a pro-oncogene and related to development and carcinogenesis [27]. In addition, these two proteins and some other proteins, such as fibroblast growth factors (FGF), fibroblast growth factor receptors (FGFR), and epidermal growth factor (EGF), are involved in cancers. The FGFR2 and FGFR3 are the cancer-related genes and top-rank proteins with the number of annotated diseases (i.e., 14 and 13 diseases recorded in OMIM, respectively). These mutations with ~ 50% probability are located at the contacting residues and have high-risk to induce diseases. Interestingly, EGFR and HRAS are key signal transporters and consistently mutated on contacting residues. Fig. 1c shows the series of protein kinases (MAPK3 and MAPK1) which are highly related to cancer by involving diverse biological functions and critical pathways such as cell growth, adhesion, survival, and differentiation [28, 29]. In addition, the RAF and BRAF, which regulate MAPKKK of ERK pathway, act as a regulatory link between the upstream signal proteins (e.g., membrane-associated Ras GTPases (i.e., KRAS, NRAS, and HRAS) and non-receptor protein tyrosine kinase (e.g., SRC) and the MAPK/ERK cascade. Furthermore, SRC can be activated by the EGFR and ERBB2 in the ERBB signal pathway for adhesion and migration [30].

According to the studies of these three microarray datasets (i.e., lung cancer [18], breast cancer [19], and brain cancer [20]) and other relevant studies, we found some particular genes expressed and mutated in specific cancer. In lung cancer dataset, some proteins (e.g., EGFR, ERBB2, BRAF, KDR, and KRAS) were indicated to have the higher level of mutation and expression in clinical samples of cancer. In breast cancer dataset, ERBB2 and EGFR were highly correlated to carcinogenesis process. These two cancers related proteins were well studied in the past and showed high probability to mutate on contacting residues in EGFR (FGFR)-MAPK pathway. Moreover, EGFR participates the metastasis mechanism of the tumor from breast tissue to brain tissue. In GBM dataset, Wnt signaling pathway plays an important role to induce tumor and has possible crosstalk with EGFR (FGFR)-MAPK pathway [31]. They affect the signaling pathways related to cell survival and growth. Furthermore, the EGFR (FGFR)-MAPK pathway of signal transduction is located at the central region of human structurally resolved PPI network (Fig. 1b). Through this pathway, the introduced signal will be spread to downstream proteins around the EGFR (FGFR)-MAPK pathway and induce numerous biological processes. Based on the mutation number and mutation site of disease, we could easily find the important proteins and pathways related to disease, such as signal transduction in cancer development.

### 3D–binding models for FGF2-FGFR2 association

FGF is a signal protein and interacts with some transmembrane receptors (e.g., FGFR1, FGFR2, FGFR3, and FGFR4) to regulate key biological processes, such as cell proliferation, survival, migration, and differentiation both during development and in the adult [32]. FGFR2 mutation can cause endometrial cancer (S252 W) or Pfeiffer syndrome (D321A) [33, 34]. Based on the previous study, S252 W mutation is the most common FGFR2 mutation [35]. According to the FGFR2-FGF2 binding interface of the structural template (PDB code: 1ev2 [36]), the S252 and D321 are the contacting residues of FGFR2 on the FGF2-FGFR2 binding interface (Fig. 1d). According to the multiple sequence alignment (MSA) results, the S252 forms a conserved van der Waals interaction to the L98 of FGF2 according to the PPI family of this template, and the D321 forms a hydrogen-bond interaction with the Q56. Because the FGF2-FGFR2 is the upstream interaction of MAPK3 and MAPK1 (ERK pathway), this mutation (i.e. S252 W) can influence the cell proliferation and apoptosis in the ERK pathway in endometrial cancer. In addition, the interacting residues S252 and L98 are conserved across three vertebrate PPI networks. These results suggest that our hDiSNet is useful for studying mutations associated with disease-related proteins.

### Network analysis of the reconstructed human structural network

To further investigate the biological meaning of our network (hDiSNet), the Gene Ontology enrichment analysis was carried out. The proteins involved in the similar process and located in similar cellular component would be the neighbors in the PPI network. We identified six cellular components (i.e., nucleus, cytoskeleton, cytoplasm, membrane, extracellular space, and others) in the human network based on the CC annotations (Additional file 1: Figure S3A). In addition, we also identified eight biological processes (i.e., cell cycle process, nucleic acid metabolic process, protein metabolic process, transport, signal transduction, proteolysis, organelle organization, and others) based on the BP annotations (Additional file 1: Figure S3B). According to these GO annotations, the reconstructed human structural interactome (hDiSNet) could be grouped into nine major regions and perform cellular functions, including cell cycle processes; nucleic acid metabolic process (e.g., transcription); cellular protein metabolic process (e.g., translation); cytoplasm signal transduction process; membrane signal transduction process; transport process; proteolysis process; extracellular proteolysis process; organelle organization (Fig. 1b).

The reconstructed human structural interactome (hDiSNet) can be used to describe the communication between the cellular protein metabolic processes (Fig. 1c). The membrane signal transduction (e.g. EGFR, FGFR, and other membrane receptors) could receive the signals from the extracellular factors and transfer the signals to the cytoplasm part signal transduction (e.g. Ras and its downstream substrate). Then, the signals could be used to activate or inactivate the cellular protein metabolic processes. The cellular protein metabolic process (brown) communicates with the cytoskeletal part (organelle organization, blue), cell cycle process (pink) and nucleic acid metabolic process (red) (Additional file 1: Figure S3B). The cytoskeletal part (organelle organization) is related to the assembly, arrangement of constituent parts, or disassembly of an organelle within a cell. The cell cycle process and nucleic acid metabolic processes are the kernel processes of a living cell. In addition, several cyclins (e.g. G1/S-specific cyclin-D2 and G2/mitotic-specific cyclin-B1) and cyclin-dependent kinases (e.g. CDK2 and CDK4) control the cell cycle and play essential roles of meiosis in cell cycle process during meiosis. The cytoplasm part transport process performs the function in the cytoplasm and communicates with the cytoplasm part signal transduction and cytoplasmic proteolysis (Additional file 1: Figure S3). The extracellular proteolysis only communicates with cellular protein metabolic process and located on the peripheral portion. These results imply that the biological behaviors of hDiSNet are consistent with known processes of a living cell.

### Disease-related mutations in hDiSNet

Disease-related mutations can be roughly classified into two broad categories (i.e., in-frame and truncating mutations) [4]. Here, the in-frame mutations are considered as missense point mutations and the in-frame insertions or deletions are likely to produce full-length proteins with local defects. In-frame mutations can lead to loss of interactions [37]. To evaluate the relationships between mutations and their associated diseases in hDiSNet, we first identified the positions of the disease-associated in-frame mutations on the corresponding proteins. In this paper, we collected 5843 in-frame mutations on 776 proteins, annotated with “OMIM-curated-records” from the dbSNP database, in hDiSNet. Next, the proteins are assigned with the interacting domains (i.e. Pfam [38] and SCOP [39]) based on the structural templates. Among the 5843 in-frame mutations, 2330 and 2090 mutations are located at 403 and 345 proteins with the Pfam and SCOP domain annotations, respectively (Additional file 1: Table S3). We found that 1653 and 1646 mutations are located at the interacting domains (i.e. Pfam and SCOP, respectively). The odds ratios of in-frame mutations involved in Pfam (3.05) and SCOP (3.34) domains are enriched than the non-interacting domain of Pfam (0.33) and SCOP (0.30), respectively (Fig. 3a and Additional file 1: Table S3). To further investigate the association between somatic mutations and their corresponding cancers in human structural interactome, we collected 42,688 somatic mutations in 266 cancer genes from COSMIC and mapped them to our hDiSNet. We then observed that 10,415 and 11,892 somatic mutations occurred in the interacting domains of Pfam and SCOP, respectively. Similarly, the results showed that the odds ratios of somatic mutations involved in Pfam (1.29) and SCOP (1.22) domains are higher than non-interacting domain regions (Additional file 1: Figure S4). These results imply that the mutations occurred in interacting domains are more related to the disease (e.g., cancer) than non-interacting domains.

In addition, we also assigned the contacting residues of proteins based on the contacting residues of the structural templates. To investigate the relationship between the mutations and contacting residues, we collected 283 proteins which have in-frame mutations on the contacting residues. Based on these 283 proteins, we found that 790 and 1426 mutations are located at the contacting and non-contacting residues, respectively. According to the odds ratio, the disease-related mutations are significantly associated with the contacting residues (2.45) comparing to non-contacting residues (0.41; Fig. 3a and Additional file 1: Table S3). We also found that somatic mutations are easier to occur in contacting residues (odds ratio: 1.42) than non-contacting residues (0.70; Additional file 1: Figure S4). According to our knowledge, the residues which form the hydrogen bonds or are conserved in PPI families may be the critical residues in the binding site and provide a higher experimental free energy [7, 16]. There are 368 (46.5%) and 468(60.8%) of 790 mutations on the contacting residues forming the hydrogen bonds and conserved in the PPI families, respectively. Conversely, 197 (24.9%) of 790 mutations neither are conserved residues and nor involved in hydrogen bonds. Fig. 3b shows the odds ratio of in-frame mutations in the contacting residues or other residues that form the hydrogen bond or is conserved in PPI families. These results indicate that the disease-related mutations are usually located at the contacting residues to form the hydrogen bonds or are conserved in the PPI families.

To further validate the association between residues and their binding affinity on binding interfaces, we collected 869 residues with experimental binding affinity annotations within 56 structural complexes derived from ASEDB and SKEMPI [40, 41]. Here, we used 786 residues in our analysis. We observed that the binding affinity (i.e. ddG) of contacting residues (1.27) is higher than non-contacting residues (0.82; *p*-value = 8.51E-05 by *U*-test) (Fig. 4a). The 116 out of 208 residues (56%) with ddG > 1.5 are preferred to play as the contacting residues (*p*-value = 3.95E-06 by Fisher’s exact test) (Fig. 4b). Moreover, we found that the contacting residues located at the domains (e.g., Pfam domains) have higher binding affinity (1.31) than non-contacting residues that outside of domains (0.70; *p*-value = 5.03E-07 by *U*-test) (Additional file 1: Figure S5A). The 81 contacting and domain residues with ddG > 1.5 have higher potential as hot spots in binding interfaces (*p*-value = 1.03E-06 by Fisher’s exact test; Additional file 1: Figure S5B). These results suggest that the binding affinity on binding interfaces could be influenced when mutations occurred in our identified contacting residues.

### hDiSNet for describing mutations in breast cancer

Breast cancer is one of the major diseases in women worldwide, with ~ 1.38 million new cases and ~ 458,000 deaths in 2008 alone [42]. Metastasis is the principal cause of death in patients with cancers [43]. Moreover, understanding of the molecular basis for breast cancer metastasis to the brain is incomplete [19]. To explore the mechanisms of breast cancer metastasis to the brain, the proteins involved in breast cancer (e.g. androgen receptor (AR); red boxes) and metastasis (e.g. Nuclear receptor subfamily 3 group C member 2 (NR3C2); yellow triangles), recorded in OMIM and provided by Bos et al. [19], are highlighted in our network, respectively (Fig. 5). According to the breast cancer- and metastasis-related proteins in our hDiSNet, we found that these proteins are located in five regions, including cytoplasm part signal transduction, cytoplasmic proteolysis, cellular protein metabolic process, nucleic acid metabolic process, and nuclear part cell cycle (Fig. 5a).

The AR is a ligand-dependent factor, which transcriptional activity is mediated by interaction with multiple co-activators [44], and is involved in the nucleic acid metabolic process (Fig. 5a). The NR3C2 is a member of the nuclear receptor superfamily, which acts as a ligand-dependent transcription factor, mediating aldosterone effects on a variety of target tissues, such as the cardiovascular and central nervous systems, and brown adipose tissue [45]. In addition, the NR3C2 has been considered to mediate breast cancer metastasis to the brain [19]. In clinical use, the progesterone receptor (PGR) is one of the important biomarkers in breast cancer [46]. In our hDiSNet, the proteins, AR, NR3C2, and PGR, located in the same sub-network (called AR-regulated sub-network) (Fig. 5b). Among 12 proteins in AR-regulated sub-network, we found that five proteins (e.g. AR, Retinoic acid receptor RXR-alpha (RXRA), and PGR) are cancer-related proteins [17], such as breast and prostate cancer. Moreover, the mutations of the other seven proteins in AR-regulated sub-network are considered as disease-association (e.g. Glucocorticoid resistance, breast cancer, and Progesterone resistance) in OMIM (Fig. 5b). In AR-regulated sub-network, nine PPIs and 11 PPIs of 41 PPIs are recorded in five databases (i.e. IntAct, MIPS, DIP, MINT, and BioGRID) and Human Protein Reference Database (HPRD) [47], which is a specific PPI database for collecting human protein-protein interactions, respectively. These 41 PPIs also share the similar GO annotations of BP (e.g., steroid hormone mediated signaling pathway and transcription from RNA polymerase II promoter) and CC (e.g., nucleoplasm) which are related to cell proliferation (Additional file 1: Table S4).

In comparison with the PPI network derived from experimental PPIs, our hDiSNet provided the clues to reveal that the mutations of target proteins affect the binding between two proteins. According to the structural template (PDB code: 3 h52 [48]), the contacting residues of A chain (orange) were I539, L544, A546, R558, T562, Q571, and D626 (Fig. 5c). The D chain (blue) were L544, Y548, L566, and A580. Based on the MSA results of A chain, we observed that the D626 was conserved residue across multiple species, and was mapped to D832 of the NR3C2 protein (Fig. 5d). The L544 on A chain could map to V750 on NR3C2. In addition, the Y548 and L566 on D chain were aligned to H689 and L707 on AR, respectively. The mutated AR has been proposed to causes breast, prostate cancer or androgen insensitivity [49, 50]. On the interface of 3h52AD, we found that the H689 of AR may form a hydrogen-bond interaction with the D832 of NR3C2 according to the PPI family, and the L707 of AR forms a conserved van der Waals interaction to the V750 of NR3C2 (Fig. 5c). Rosa et al. analyzed the structural consequences of the H689P mutation, and they suggested that it likely to perturb the conformation of the second helix of the AR ligand-binding domain, which contains the residues critical for androgen binding [51]. In addition, sequencing identified a point mutation in exon 4 that is responsible for a CTG to CGG replacement (L707R), which is located at the amino-terminal part of the AR ligand-binding domain [52]. Therefore, the present results suggest that our hDiSNet is useful for understanding the influences of mutations on binding interfaces in diseases.

### hDiSNet for exploring somatic mutations in brain cancer

The grade IV astrocytomas (i.e., GBM) are categorized as an aggressive class of brain cancer, which involved in hallmark characteristics of proliferation, necrosis, genetic instability, and chemoresistance [53]. In clinical studies, it is difficult to treat and has a poor prognosis when patients were diagnosed with the GBM, who have a poor median overall survival of 12 months and 2-year survival rates were less than 10% [54]. Multiple histopathological and genetic reports have recognized the epidermal growth factor receptor (EGFR) and its downstream signaling pathways as commonly dysregulated elements in the GBM tumors [55–57]. Recently, some studies have observed that mutations of ErbB family receptors (e.g., EGFR) and downstream proteins may mediate the GBM tumorigenesis [54, 58, 59]. To explore the association of signaling pathways and somatic mutations in GBM, we collected the proteins of the ErbB signaling pathway (hsa04012) from KEGG pathway [28] and mapped somatic mutations from COSMIC into our hDiSNet. Based on our hDiSNet, the sub-network contains 41 proteins and 128 PPIs, and 18 out of 41 proteins are annotated with 3577 somatic mutations (Additional file 1: Figure S6A). We found that 18 proteins involved in more than 3 different primary tissue (cancer) types associated with somatic mutations, such as EGFR, ERBB2, and ABL1. In ErbB-regulated sub-network, 75 (59%) and 53 (41%) of 128 PPIs are derived from human and non-human templates, respectively, and total 92 (72%) PPIs are recorded in five databases. In addition, EGFR has 10 interacting partners and they share similar biological functions, such as EGFR-ERBB2 (RSS-BP 0.93) participated in cell surface receptor signaling pathway and EGFR-ABL1 (RSS-BP 0.93) involved in regulation of cell cycle (Additional file 1: Table S5). Moreover, we observed that EGFR has 732 somatic mutations across 22 different primary cancer types, of these 732 mutations, 14 mutations are found in GBM patients (e.g., Y270C and R149W). Based on our PPI families, we found that 131 out of 732 EGFR mutations (18%) occurred in binding interfaces, such as Y270C. The importance of EGFR Y270 was reported as the phosphorylation site in PhosphoSitePlus database [60] and identified by small-scale experiments [61].

According to the structural template (PDB code: 1ivo [62]), the contacting residue of A chain (cyan) was Y246, and B chain (purple) was G264 and C283 (Additional file 1: Figure S6B). Based on the MSA results of A chain, we observed that the Y246 was conserved residue across multiple species, and mapped to the homologous protein EGFR was Y270 (Additional file 1: Figure S6C). The interacting residues of Y246 were G264 and C283 in B chain of structural template 1ivo, which aligned to the homologous protein ERBB2 was G292 and C311. On the interface of EGFR-ERBB2 (1ivoAB), we found that the Y270 of EGFR (Y246 in A chain) may form the conserved hydrogen-bond interaction with the G292 and C311 of ERBB2 (G264 and C283 in B chain; Additional file 1: Figure S6B). In addition, the EGFR-Y270, ERBB2-G292, and ERBB2-C311 are not only contacting residues, but also located at the furin-like domain (Pfam ID: PF00757) of extracellular regions. Previous studies have indicated that mutations occurred in extracellular domains 2 and 4 of EGFR could disrupt auto-inhibitory contact regions and promote out of control ligand-independent receptor activation in cancer [63]. Furthermore, the genomic mutations of EGFR have been observed to affect the extracellular domain regions, for instance, EGFR-Y270 mutation affects extracellular domains 2 [64] and confers oncogenicity in GBM [65]. The results show that our proposed human structural interactome (hDiSNet) is powerful for investigating the effects of somatic mutations on binding interfaces and revealing the causes of disease.

## Conclusions

We have reconstructed a human structural interactome (hDiSNet) with detailed atomic binding models and disease-associated mutations by enhancing our PPI families and 3D–domain interologs. Our hDiSNet expands the number of PPIs compared to present structural PPI network. The experimental results show that our predicted PPIs have significantly consistent GO annotations and high co-expression correlations. Our hDiSNet provides the insights into human genetic disease and their associated proteins and mutations, such as the structural FGFR (EGFR)-MAPK pathway for interpreting the breast cancer and ErbB sub-network in brain cancer. Our results show that the disease-related mutations are often located at the contacting residues forming the hydrogen bonds and are conserved in the PPI family. Furthermore, the binding affinity analysis indicates our identified contacting residues have high potential as hot spots. We believe that our method is a useful tool to reconstruct structural interactome with detailed atomic interactions to examine the linkages between the diseases and mutations and to interpret structural genomics and disease associations.

## Additional file

Additional file 1: Supplementary Material. (DOCX 2898 kb)

## Abbreviations

AR: Androgen receptor
BP: Biological process
CC: Cellular component
CGC: Cancer Gene Census
COSMIC: Catalogue Of Somatic Mutations In Cancer
dbSNP: Database of single nucleotide polymorphisms
EGFR: Epidermal growth factor receptor
GBM: Glioblastoma multiforme
GEO: Gene Expression Omnibus
GO: Gene Ontology
HC: High-confidence
hDiSNet: Human three-dimensional structural PPI network
HPRD: Human Protein Reference Database
hSIN: Human structural interaction network
IEXP: Integrated experimental
MF: Molecular function
NR3C2: Nuclear receptor subfamily 3 group C member 2
OMIM: Online Mendelian Inheritance in Man
PDB: Protein Data Bank
PGR: Progesterone receptor
PPI: Protein-protein interactions
RSS: Relative specificity similarity
RXRA: RXR-alpha
