# Supplementary material for: A homologous mapping method for three-dimensional reconstruction of protein networks reveals disease-associated mutations
Source: BMC Syst Biol. 2018 Mar 19;12(Suppl 2):13. doi: 10.1186/s12918-018-0537-2 (PMC5861491; doi:10.1186/s12918-018-0537-2)
Supplement: Supplementary file 1 — Supplementary Material. (DOCX 2898 kb) [file 12918_2018_537_MOESM1_ESM.docx]

**A homologous mapping method for three-dimensional reconstruction of protein networks reveals disease-associated mutations**

**(Supplementary Material)**

Sing-Han Huang^1^, Yu-Shu Lo^1^, Yong-Chun Luo^1^, Yu-Yao Tseng^1^, and Jinn-Moon Yang^1,2*^

^1^Institute of Bioinformatics and Systems Biology, National Chiao Tung University, Hsinchu, 30050, Taiwan

^2^ Department of Biological Science and Technology, National Chiao Tung University, Hsinchu, 30050, Taiwan

**Table of Contents**

**I. Additional Figures**

Additional Figure S1. The co-expression correlations of PPIs derived from PPI families and experimental data sets.

Additional Figure S2. The node degree distributions of different PPI networks.

Additional Figure S3. Characteristics of hDiSNet using GO annotations.

Additional Figure S4. The enrichment of our predicted interacting domains and contacting residues by using 42,688 mutations in 266 cancer genes from COSMIC.

Additional Figure S5. The binding affinity association with contacting residues in domains and non-contacting residues in non-domain regions.

Additional Figure S6. The ErbB signaling pathway in hDiSNet for brain cancer.

**II. Additional Tables**

Additional Table S1. The mutations on AR and the proteins in EGFR (FGFR)-MAPK pathway, and their corresponding disease.

Additional Table S2. The number of proteins and PPIs in five public databases.

Additional Table S3. The statistical results of the in-frame mutations involved in interacting domain (i.e. SCOP, Pfam) and contacting residues.

Additional Table S4. 41 PPIs in the sub-network of AR and NR3C2.

Additional Table S5. 128 PPIs in the ErbB sub-network.

**I. Additional Figures**

## Additional Figure S1. The co-expression correlations of PPIs derived from PPI families and experimental data sets.

The gene co-expressions of seven PPI data sets are derived from three publications, including (A) lung adenocarcinomas (GSE12667); (B) Primary breast tumors (GSE12276) (C) glioblastoma (GSE7696). The PPI sets include all protein pairs (dot black), our predicted PPIs derived from human (gray) and non-human (green) templates, IEXP (blue) and HC (red) experimental PPIs, overlapped PPIs between our hDiSNet and IEXP PPIs (yellow), and hSIN (purple). The overlapped PPIs have similar co-expression correlation to hSIN, and our inferred PPIs have significantly higher correlation (p-value < 0.001, Mann–Whitney *U* test) than all protein pairs.

## Additional Figure S2. The node degree distributions of different PPI networks.

The degree exponent γ value is (A) 2.05 in our hDiSNet, (B) 2.23 in hSIN, (C) 1.48 in HC, and (D) 1.43 in IEXP. According to the typical scale-free network properties, the degree exponent is usually within 2 ≤ γ ≤ 3. Our hDiSNet is a scale-free network.

## Additional Figure S3. Characteristics of hDiSNet using GO annotations.

(A) According to GO cellular component (CC) annotations, the proteins in the hDiSNet could be grouped into nine groups with five CC annotations, including 269 proteins in nuclear part (GO:0044428, red), 704 proteins in intracellular region (GO:0005737, yellow), 232 proteins in cytoskeletal part (GO:0044430, green), 338 proteins in membrane (GO: 0016021 and GO:0005886, purple), 202 proteins in extracellular space (GO:0005615, blue). (B) Based on GO biological processes (BP) annotations, 134 proteins are annotated with nucleic acid metabolic process (e.g., transcription) (GO:0090304, red); 189 proteins are annotated with regulation of cell cycle (GO:0051726, pink); 476 proteins are annotated with cellular protein metabolic process (e.g., translation) (GO:0044267, orange); 333 proteins are with signal transduction (GO:0007165, cyan); 204 proteins are with transport (GO:0015031, green); 287 proteins are with proteolysis (GO:0006508, yellow); 59 proteins are with organelle organization (GO:000996, blue).

## Additional Figure S4. The enrichment of our predicted interacting domains and contacting residues by using 42,688 mutations in 266 cancer genes from COSMIC.

The odds ratios of somatic mutations in interacting domains (SCOP with blue and Pfam with red), contacting residues (green), non-interacting domains (SCOP with dot blue and Pfam with dot red), and non-contacting residues (dot green). The somatic mutations are usually occurred in the interacting domains and contacting residues.

## Additional Figure S5. The binding affinity association with contacting residues in domains and non-contacting residues in non-domain regions.

(A) The average ddGs of contacting residues in domains and non-contacting residues in non-domain regions are 1.31 and 0.70, respectively. (B) The ratios of contacting and non-contacting residues with more than 1.5 ddG and with less than 1.5 ddG. The p-value of the enrichment of contacting residues in domains is 1.03E-06 by Fisher’s exact test.

## Additional Figure S6. The ErbB signaling pathway in hDiSNet for brain cancer.

(A) The sub-network of the ErbB signaling pathway in our hDiSNet contains 128 structurally resolved PPIs among 41 proteins. The node size denotes the numbers of somatic mutations recorded in COSMIC. Colored nodes indicate the ratios of mutations in the contacting (orange) and non-contacting (gray) residues. The colored borders of nodes indicate the numbers of cancer types involved in mutated proteins. (B) Detailed atomic interactions of PPI EGFR-ERBB2 and the contacting residue analysis using the 3D-template complex (PDB code: 1ivo) with the furin-like domain (Pfam ID: PF00757). (C) The multiple sequence alignments of PPI family of EGFR and ERBB2 proteins. The interacting residues are colored according to their types: for forming hydrogen bonds (green), conserved (orange), both for forming hydrogen bonds and conserved (yellow), and for forming van der Waals force (gray).

**II. Additional Tables**

## Additional Table S1. The mutations on AR and the proteins in EGFR (FGFR)-MAPK pathway, and their corresponding disease.

| Gene | Uniprot  AC | Mutation | Disease |
| --- | --- | --- | --- |
| BRAF | P15056 | D594G | lymphoma, non-hodgkin, somatic |
| BRAF | P15056 | E501G | cardiofaciocutaneous syndrome 1 |
| BRAF | P15056 | E501K | cardiofaciocutaneous syndrome 1 |
| BRAF | P15056 | K601E | colorectal cancer, somatic  thyroid carcinoma, follicular, somatic, included |
| BRAF | P15056 | L597R | adenocarcinoma of lung, somatic |
| BRAF | P15056 | L597V | nonsmall cell lung cancer, somatic |
| EGFR | P00533 | G719S | nonsmall cell lung cancer, response to tyrosine kinase inhibitor in, somatic |
| EGFR | P00533 | L858R | nonsmall cell lung cancer, response to tyrosine kinase inhibitor in, somatic  adenocarcinoma of lung, response to tyrosine kinase inhibitor in, somatic, included |
| ERBB2 | P04626 | E914K | glioblastoma, somatic |
| ERBB2 | P04626 | L755P | adenocarcinoma of lung, somatic |
| FGF10 | O15520 | I156R | ladd syndrome |
| FGF3 | P11487 | Y49C | deafness, congenital, with inner ear agenesis, microtia, and microdontia |
| FGFR1 | P11362 | A167S | hypogonadotropic hypogonadism 2 with anosmia |
| FGFR1 | P11362 | D768Y | hypogonadotropic hypogonadism 2 without anosmia, susceptibility to |
| FGFR1 | P11362 | L342S | hypogonadotropic hypogonadism 2 with anosmia, susceptibility to |
| FGFR1 | P11362 | N724K | hypogonadotropic hypogonadism 2 without anosmia, susceptibility to |
| FGFR1 | P11362 | P252R | apert syndrome |
| FGFR1 | P11362 | P722H | hypogonadotropic hypogonadism 2 without anosmia, susceptibility to |
| FGFR1 | P11362 | P722S | hypogonadotropic hypogonadism 2 with anosmia |
| FGFR1 | P11362 | Q764H | hypogonadotropic hypogonadism 2 without anosmia |
| FGFR1 | P11362 | W666R | hypogonadotropic hypogonadism 2 with anosmia, susceptibility to |
| FGFR2 | P21802 | A315S | craniosynostosis, nonsyndromic unicoronal |
| FGFR2 | P21802 | A344G | jackson-weiss syndrome  crouzon syndrome, included |
| FGFR2 | P21802 | D321A | pfeiffer syndrome |
| FGFR2 | P21802 | K526E | crouzon syndrome  scaphocephaly, maxillary retrusion, and mental retardation, included |
| FGFR2 | P21802 | P253R | apert syndrome |
| FGFR2 | P21802 | P253S | pfeiffer syndrome variant |
| FGFR2 | P21802 | Q289P | crouzon syndrome  jackson-weiss syndrome, included |
| FGFR2 | P21802 | S252F | apert syndrome |
| FGFR2 | P21802 | S252W | apert syndrome  endometrial cancer, somatic, included |
| FGFR2 | P21802 | S347C | crouzon syndrome |
| FGFR2 | P21802 | S354C | crouzon syndrome |
| FGFR2 | P21802 | T341P | pfeiffer syndrome |
| FGFR3 | P22607 | D513N | ladd syndrome |
| FGFR3 | P22607 | E322K | colorectal cancer, somatic |
| FGFR3 | P22607 | K650E | thanatophoric dysplasia, type ii  multiple myeloma, somatic, included spermatocytic seminoma, somatic, included |
| FGFR3 | P22607 | K650M | saddan dysplasia  thanatophoric dysplasia, type i, included |
| FGFR3 | P22607 | K650N | hypochondroplasia |
| FGFR3 | P22607 | K650Q | hypochondroplasia  bladder cancer, somatic, included |
| FGFR3 | P22607 | P250R | muenke syndrome  saethre-chotzen syndrome, included |
| FGFR3 | P22607 | R248C | thanatophoric dysplasia, type i  multiple myeloma, somatic, included skeletal dysplasia with acanthosis nigricans, included nevus, epidermal, somatic, included keratosis, seborrheic, somatic, included |
| FGFR3 | P22607 | R621H | camptodactyly, tall stature, and hearing loss syndrome |
| FGFR3 | P22607 | S249C | thanatophoric dysplasia, type i  cervical cancer, somatic, included bladder cancer, somatic, included keratosis, seborrheic, somatic, included |
| FGFR3 | P22607 | S279C | achondroplasia  hypochondroplasia, included |
| FGFR3 | P22607 | Y278C | hypochondroplasia |
| HRAS | P01112 | A146T | costello syndrome |
| HRAS | P01112 | A146V | costello syndrome |
| HRAS | P01112 | E63K | myopathy, congenital, with excess of muscle spindles |
| HRAS | P01112 | G12A | costello syndrome |
| HRAS | P01112 | G12C | costello syndrome  nevus sebaceous, somatic, included epidermal nevus, somatic, included |
| HRAS | P01112 | G12D | costello syndrome, severe  nevus sebaceous, somatic, included |
| HRAS | P01112 | G12S | costello syndrome  myopathy, congenital, with excess of muscle spindles, included epidermal nevus with urothelial cancer, somatic, included nevus sebaceous, somatic, included |
| HRAS | P01112 | G12V | bladder cancer, somatic  costello syndrome, included myopathy, congenital, with excess of muscle spindles, included epidermal nevus, somatic, included |
| HRAS | P01112 | G13C | costello syndrome |
| HRAS | P01112 | G13D | costello syndrome |
| HRAS | P01112 | K117R | costello syndrome |
| HRAS | P01112 | Q22K | myopathy, congenital, with excess of muscle spindles |
| HRAS | P01112 | Q61K | schimmelpenning-feuerstein-mims syndrome, somatic mosaic |
| HRAS | P01112 | T58I | costello syndrome |
| KDR | P35968 | P1147S | hemangioma, capillary infantile, somatic |
| KRAS | P01116 | A59T | bladder cancer, transitional cell, somatic |
| KRAS | P01116 | G12C | lung cancer, somatic |
| KRAS | P01116 | G12D | pancreatic carcinoma, somatic  gastric cancer, somatic, included epidermal nevus, somatic, included nevus sebaceous, somatic, included schimmelpenning-feuerstein-mims syndrome, somatic mosaic, included juvenile myelomonocytic leukemia, somatic, included ras-associated autoimmune leukoproliferative disorder, somatic, included |
| KRAS | P01116 | G12R | lung cancer, squamous cell, somatic  bladder cancer, somatic, included |
| KRAS | P01116 | G12S | gastric cancer, somatic  juvenile myelomonocytic leukemia, somatic, included |
| KRAS | P01116 | G12V | pancreatic carcinoma, somatic  nevus sebaceous, somatic, included |
| KRAS | P01116 | G13D | breast adenocarcinoma, somatic  juvenile myelomonocytic leukemia, somatic, included ras-associated autoimmune leukoproliferative disorder, somatic, included |
| KRAS | P01116 | G13R | pilocytic astrocytoma, somatic |
| KRAS | P01116 | G60R | cardiofaciocutaneous syndrome 2 |
| KRAS | P01116 | G60S | noonan syndrome 3 |
| KRAS | P01116 | P34R | cardiofaciocutaneous syndrome 2 |
| KRAS | P01116 | T58I | noonan syndrome 3 |
| KRAS | P01116 | Y71H | cardiofaciocutaneous syndrome 2 |
| NRAS | P01111 | G13D | juvenile myelomonocytic leukemia, somatic  ras-associated autoimmune leukoproliferative disorder, somatic (1 patient), included noonan syndrome 6, included |
| NRAS | P01111 | G13R | rectal cancer, somatic  melanocytic nevus syndrome, congenital, somatic, included |
| NRAS | P01111 | Q61R | thyroid carcinoma, follicular, somatic  epidermal nevus, somatic, included melanocytic nevus syndrome, congenital, somatic, included neurocutaneous melanosis, somatic, included schimmelpenning-feuerstein-mims syndrome, somatic mosaic, included |
| NRAS | P01111 | T50I | noonan syndrome 6 |
| RAF1 | P04049 | T491R | noonan syndrome 5 |
| RET | P07949 | M918T | multiple endocrine neoplasia, type iib  thyroid carcinoma, sporadic medullary, included pheochromocytoma, somatic, included |
| RET | P07949 | R897Q | hirschsprung disease, susceptibility to, 1 |
| RET | P07949 | R912P | thyroid carcinoma, familial medullary |
| RET | P07949 | R972G | hirschsprung disease, susceptibility to, 1 |
| RET | P07949 | S765P | hirschsprung disease, susceptibility to, 1 |
| AR | P10275 | A721T | prostate cancer |
| AR | P10275 | H689P | androgen insensitivity, complete |
| AR | P10275 | L707R | androgen insensitivity, complete |
| AR | P10275 | L712F | androgen insensitivity syndrome |
| AR | P10275 | R607Q | androgen insensitivity, partial, with or without breast cancer |
| AR | P10275 | R840C | androgen insensitivity syndrome |
| AR | P10275 | V730M | prostate cancer |

## Additional Table S2. The number of proteins and PPIs in five public databases.

| **Source database** | **Number of PPIs** | **Number of proteins** | **Date (version)** |
| --- | --- | --- | --- |
| **IntAct** | **278,517** | **66,521** | **Feb, 2014** |
| **MIPS** | **18,529** | **4,571** | **Oct, 2008** |
| **DIP** | **49,203** | **18,197** | **Feb, 2014** |
| **MINT** | **126,548** | **42,124** | **Feb, 2014** |
| **BioGRID** | **847,633** | **69,077** | **Feb, 2014** |
| **Non-redundant** | **1,055,701** | **103,567** |  |
| **Non-redundant human PPI** | **181,868** | **16,433** |  |
| **High-confidence human PPI** | **25,675** | **8,965** |  |

## Additional Table S3. The statistical results of the in-frame mutations involved in interacting domain (i.e. SCOP, Pfam) and contacting residues.

| Type | No. of proteins | Total sequence length | No. of type residues ^a^ | No. of non-type residues ^b^ | No. of mutation | No. of mutations on  type residue ^c^ | No. of mutations not on the type residue ^d^ | Odds ratio |
| --- | --- | --- | --- | --- | --- | --- | --- | --- |
| Pfam domain | 403 | 282,517 | 125,606 | 156,911 | 2,330 | 1,653 | 677 | 3.05 |
| SCOP  domain | 345 | 214,674 | 112,967 | 101,707 | 2,090 | 1,646 | 444 | 3.34 |
| Contacting residue | 283 | 176,266 | 32,522 | 143,744 | 2,216 | 790 | 1426 | 2.45 |

^a^ No. of type residues: The sums of residues which locate on the Pfam and SCOP domains and are the contacting residues, respectively.

^b^ No. of non-type residues = Total sequence length - No. of type residues.

^c^ No. of mutations on type residue: The number of mutations which residues locate on the Pfam and SCOP domains and are the contacting residues, respectively.

^d^ No. of mutations not on the type residue = No. of mutation - No. of mutations on type residue

## Additional Table S4. 41 PPIs in the sub-network of AR and NR3C2.

| Protein 1 | Protein 2 | Gene name 1 | Gene name 2 | RSS of BP | RSS of CC | PPI database^*1^ | HPRD |
| --- | --- | --- | --- | --- | --- | --- | --- |
| P10275 | P49116 | AR | NR2C2 | 0.917 | 0.875 | Y | Y |
| P41235 | P10275 | HNF4A | AR | 0.923 | 0.923 | Y | Y |
| P41235 | P49116 | HNF4A | NR2C2 | 0.917 | 0.875 | Y | Y |
| P13056 | P10275 | NR2C1 | AR | 0.917 | 0.875 | Y | Y |
| P04150 | P08235 | NR3C1 | NR3C2 | 0.917 | 0.875 | Y | Y |
| P04150 | P28702 | NR3C1 | RXRB | 0.923 | 0.875 | Y | Y |
| P08235 | P19793 | NR3C2 | RXRA | 0.917 | 0.875 | Y | Y |
| P28702 | P49116 | RXRB | NR2C2 | 0.917 | 0.875 | Y | Y |
| P49116 | P13056 | NR2C2 | NR2C1 | 0.917 | 0.875 | Y | Y |
| P04150 | P10275 | NR3C1 | AR | 0.917 | 0.875 | - | Y |
| P08235 | P10275 | NR3C2 | AR | 0.917 | 0.875 | - | Y |
| P10275 | P19793 | AR | RXRA | 0.917 | 0.875 | - | - |
| P10275 | P28702 | AR | RXRB | 0.917 | 0.875 | - | - |
| P10275 | P48443 | AR | RXRG | 0.917 | 0.875 | - | - |
| O95718 | P62508 | ESRRB | ESRRG | 0.917 | 0.875 | - | - |
| O95718 | P19793 | ESRRB | RXRA | 0.917 | 0.875 | - | - |
| O95718 | P28702 | ESRRB | RXRB | 0.917 | 0.875 | - | - |
| O95718 | P48443 | ESRRB | RXRG | 0.917 | 0.875 | - | - |
| P62508 | P19793 | ESRRG | RXRA | 0.917 | 0.875 | - | - |
| P62508 | P28702 | ESRRG | RXRB | 0.917 | 0.875 | - | - |
| P62508 | P48443 | ESRRG | RXRG | 0.917 | 0.875 | - | - |
| P13056 | P19793 | NR2C1 | RXRA | 0.923 | 0.875 | - | - |
| P13056 | P28702 | NR2C1 | RXRB | 0.917 | 0.875 | - | - |
| P13056 | P48443 | NR2C1 | RXRG | 0.917 | 0.875 | - | - |
| P04150 | O95718 | NR3C1 | ESRRB | 1 | 0.875 | - | - |
| P04150 | P62508 | NR3C1 | ESRRG | 0.923 | 0.875 | - | - |
| P04150 | P06401 | NR3C1 | PGR | 0.917 | 0.875 | - | - |
| P08235 | O95718 | NR3C2 | ESRRB | 0.923 | 0.875 | - | - |
| P08235 | P62508 | NR3C2 | ESRRG | 0.917 | 0.875 | - | - |
| P08235 | P06401 | NR3C2 | PGR | 0.917 | 0.875 | - | - |
| P06401 | P10275 | PGR | AR | 0.917 | 0.875 | - | - |
| P06401 | O95718 | PGR | ESRRB | 0.917 | 0.875 | - | - |
| P06401 | P62508 | PGR | ESRRG | 0.917 | 0.875 | - | - |
| P19793 | P41235 | RXRA | HNF4A | 0.917 | 0.875 | - | - |
| P19793 | P49116 | RXRA | NR2C2 | 0.917 | 0.875 | - | - |
| P19793 | P28702 | RXRA | RXRB | 0.917 | 0.875 | - | - |
| P19793 | P48443 | RXRA | RXRG | 0.923 | 0.875 | - | - |
| P28702 | P41235 | RXRB | HNF4A | 0.923 | 0.875 | - | - |
| P48443 | P41235 | RXRG | HNF4A | 0.917 | 0.875 | - | - |
| P48443 | P49116 | RXRG | NR2C2 | 0.923 | 0.875 | - | - |
| P48443 | P28702 | RXRG | RXRB | 0.917 | 0.875 | - | - |

## Additional Table S5. 128 PPIs in the ErbB sub-network.

| Protein 1 | Protein 2 | Gene name 1 | Gene name 2 | RSS of BP | RSS of CC | PPI database |
| --- | --- | --- | --- | --- | --- | --- |
| P28482 | P36507 | MAPK1 | MAP2K2 | 1 | 1 | Y |
| P27361 | Q02750 | MAPK3 | MAP2K1 | 1 | 1 | Y |
| P28482 | P28482 | MAPK1 | MAPK1 | 1 | 1 | Y |
| P28482 | P27361 | MAPK1 | MAPK3 | 1 | 1 | Y |
| Q15303 | Q15303 | ERBB4 | ERBB4 | 1 | 0.9 | Y |
| P27361 | P36507 | MAPK3 | MAP2K2 | 1 | 1 | Y |
| Q15303 | P42684 | ERBB4 | ABL2 | 0.9 | 0.875 | Y |
| Q15303 | P21860 | ERBB4 | ERBB3 | 0.9 | 0.8 | Y |
| P27361 | P27361 | MAPK3 | MAPK3 | 1 | 1 | Y |
| Q15303 | P04626 | ERBB4 | ERBB2 | 0.9 | 0.875 | Y |
| Q15303 | P00533 | ERBB4 | EGFR | 0.9 | 0.875 | Y |
| Q15303 | P00519 | ERBB4 | ABL1 | 0.9 | 0.875 | Y |
| P42684 | P00519 | ABL2 | ABL1 | 0.929 | 0.889 | Y |
| Q13177 | Q13177 | PAK2 | PAK2 | 1 | 0.875 | Y |
| P31749 | P46527 | AKT1 | CDKN1B | 0.9 | 0.875 | Y |
| Q13177 | P15056 | PAK2 | BRAF | 0.875 | 0.875 | Y |
| Q13177 | P04049 | PAK2 | RAF1 | 0.929 | 0.875 | Y |
| Q13177 | P00519 | PAK2 | ABL1 | 0.929 | 0.875 | Y |
| P21860 | P21860 | ERBB3 | ERBB3 | 1 | 1 | Y |
| P21860 | P04626 | ERBB3 | ERBB2 | 0.9 | 0.857 | Y |
| P21860 | P01133 | ERBB3 | EGF | 0.9 | 0.8 | Y |
| P17252 | P49841 | PRKCA | GSK3B | 0.9 | 0.875 | Y |
| P04626 | Q13153 | ERBB2 | PAK1 | 0.929 | 0.875 | Y |
| P04626 | P42684 | ERBB2 | ABL2 | 0.929 | 0.771 | Y |
| P04626 | P04626 | ERBB2 | ERBB2 | 1 | 0.9 | Y |
| P04626 | P00519 | ERBB2 | ABL1 | 0.929 | 0.875 | Y |
| P00533 | P42684 | EGFR | ABL2 | 0.929 | 0.771 | Y |
| P00533 | P21860 | EGFR | ERBB3 | 0.9 | 0.9 | Y |
| Q05397 | Q05397 | PTK2 | PTK2 | 1 | 1 | Y |
| P00533 | P17252 | EGFR | PRKCA | 1 | 0.875 | Y |
| Q05397 | P49841 | PTK2 | GSK3B | 0.929 | 0.875 | Y |
| P00533 | P12931 | EGFR | SRC | 1 | 0.875 | Y |
| Q05397 | P12931 | PTK2 | SRC | 1 | 0.875 | Y |
| P00533 | P04626 | EGFR | ERBB2 | 0.929 | 0.9 | Y |
| P00533 | P01135 | EGFR | TGFA | 0.9 | 0.875 | Y |
| P00533 | P01133 | EGFR | EGF | 1 | 0.818 | Y |
| P00533 | P00533 | EGFR | EGFR | 1 | 1 | Y |
| P00533 | P00519 | EGFR | ABL1 | 0.929 | 0.875 | Y |
| Q05397 | P04626 | PTK2 | ERBB2 | 0.929 | 0.875 | Y |
| Q05397 | P00533 | PTK2 | EGFR | 1 | 0.875 | Y |
| P49841 | P49841 | GSK3B | GSK3B | 1 | 1 | Y |
| P49841 | P31751 | GSK3B | AKT2 | 0.9 | 0.875 | Y |
| P49841 | P31749 | GSK3B | AKT1 | 0.917 | 0.875 | Y |
| P45985 | P45985 | MAP2K4 | MAP2K4 | 1 | 0.875 | Y |
| P45985 | P31749 | MAP2K4 | AKT1 | 0.875 | 0.875 | Y |
| P45985 | O14733 | MAP2K4 | MAP2K7 | 1 | 0.875 | Y |
| P00519 | P00519 | ABL1 | ABL1 | 1 | 0.889 | Y |
| P15056 | P31749 | BRAF | AKT1 | 0.917 | 0.875 | Y |
| P15056 | P15056 | BRAF | BRAF | 0.923 | 0.875 | Y |
| P23443 | P31749 | RPS6KB1 | AKT1 | 0.909 | 0.875 | Y |
| P15056 | P04049 | BRAF | RAF1 | 0.923 | 0.875 | Y |
| P10398 | Q02750 | ARAF | MAP2K1 | 0.923 | 0.875 | Y |
| P10398 | P36507 | ARAF | MAP2K2 | 0.923 | 0.875 | Y |
| P10398 | P15056 | ARAF | BRAF | 0.923 | 0.875 | Y |
| P10398 | P04049 | ARAF | RAF1 | 0.923 | 0.875 | Y |
| P04049 | Q02750 | RAF1 | MAP2K1 | 0.929 | 0.875 | Y |
| P04049 | P36507 | RAF1 | MAP2K2 | 0.929 | 0.875 | Y |
| P04049 | P31749 | RAF1 | AKT1 | 0.917 | 0.875 | Y |
| P04049 | P12931 | RAF1 | SRC | 0.929 | 0.875 | Y |
| P04049 | P04049 | RAF1 | RAF1 | 1 | 0.917 | Y |
| P12931 | Q9UBS0 | SRC | RPS6KB2 | 0.9 | 0.875 | Y |
| P12931 | Q13177 | SRC | PAK2 | 0.929 | 0.875 | Y |
| P12931 | P46527 | SRC | CDKN1B | 0.9 | 0.875 | Y |
| P16333 | P62993 | NCK1 | GRB2 | 1 | 0.875 | Y |
| P12931 | P42684 | SRC | ABL2 | 0.929 | 0.875 | Y |
| P12931 | P23443 | SRC | RPS6KB1 | 0.875 | 0.875 | Y |
| P12931 | P12931 | SRC | SRC | 1 | 0.917 | Y |
| P12931 | P04626 | SRC | ERBB2 | 0.929 | 0.875 | Y |
| Q9NQU5 | P31749 | PAK6 | AKT1 | 0.875 | 0.875 | Y |
| P12931 | P00519 | SRC | ABL1 | 1 | 0.875 | Y |
| Q02750 | Q02750 | MAP2K1 | MAP2K1 | 1 | 1 | Y |
| Q02750 | P15056 | MAP2K1 | BRAF | 0.923 | 0.875 | Y |
| Q13153 | Q13177 | PAK1 | PAK2 | 0.929 | 0.875 | Y |
| Q13153 | Q13153 | PAK1 | PAK1 | 1 | 1 | Y |
| Q13153 | P31749 | PAK1 | AKT1 | 0.917 | 0.875 | Y |
| Q13153 | P04049 | PAK1 | RAF1 | 0.929 | 0.875 | Y |
| P01116 | P04049 | KRAS | RAF1 | 0.929 | 0.875 | Y |
| P36507 | Q02750 | MAP2K2 | MAP2K1 | 1 | 1 | Y |
| P36507 | P36507 | MAP2K2 | MAP2K2 | 1 | 1 | Y |
| P36507 | P15056 | MAP2K2 | BRAF | 0.923 | 0.875 | Y |
| P45984 | P45985 | MAPK9 | MAP2K4 | 1 | 0.875 | Y |
| P62993 | P62993 | GRB2 | GRB2 | 1 | 1 | Y |
| P01112 | Q07889 | HRAS | SOS1 | 0.929 | 0.875 | Y |
| P45983 | P45985 | MAPK8 | MAP2K4 | 1 | 0.875 | Y |
| P45983 | P23443 | MAPK8 | RPS6KB1 | 0.875 | 0.875 | Y |
| P62993 | P42684 | GRB2 | ABL2 | 0.929 | 0.875 | Y |
| P01112 | P15056 | HRAS | BRAF | 0.923 | 0.875 | Y |
| P01112 | P10398 | HRAS | ARAF | 0.923 | 0.875 | Y |
| P45983 | O14733 | MAPK8 | MAP2K7 | 1 | 0.875 | Y |
| P01112 | P04049 | HRAS | RAF1 | 0.929 | 0.875 | Y |
| P01111 | P15056 | NRAS | BRAF | 0.923 | 0.8 | Y |
| P01111 | P04049 | NRAS | RAF1 | 0.929 | 0.8 | Y |
| P28482 | Q02750 | MAPK1 | MAP2K1 | 1 | 1 | Y |
| Q15303 | P01135 | ERBB4 | TGFA | 0.9 | 0.875 | - |
| Q15303 | P01133 | ERBB4 | EGF | 0.909 | 0.8 | - |
| P42684 | P42684 | ABL2 | ABL2 | 1 | 0.889 | - |
| P21860 | P01135 | ERBB3 | TGFA | 0.9 | 0.8 | - |
| Q9UBS0 | Q9UBS0 | RPS6KB2 | RPS6KB2 | 0.9 | 0.875 | - |
| Q9UBS0 | P23443 | RPS6KB2 | RPS6KB1 | 0.9 | 0.875 | - |
| P15056 | P12931 | BRAF | SRC | 0.909 | 0.875 | - |
| P23443 | P23443 | RPS6KB1 | RPS6KB1 | 1 | 0.917 | - |
| P10398 | P12931 | ARAF | SRC | 0.875 | 0.875 | - |
| P10398 | P10398 | ARAF | ARAF | 0.923 | 0.875 | - |
| Q13153 | Q9P286 | PAK1 | PAK7 | 0.875 | 0.875 | - |
| Q13153 | Q9NQU5 | PAK1 | PAK6 | 0.875 | 0.875 | - |
| P01116 | Q9NQU5 | KRAS | PAK6 | 0.829 | 0.771 | - |
| P01116 | Q07890 | KRAS | SOS2 | 0.929 | 0.771 | - |
| P01116 | Q07889 | KRAS | SOS1 | 0.929 | 0.8 | - |
| Q13153 | O96013 | PAK1 | PAK4 | 0.875 | 0.875 | - |
| Q13153 | O75914 | PAK1 | PAK3 | 0.917 | 0.714 | - |
| P01116 | P15056 | KRAS | BRAF | 0.923 | 0.8 | - |
| P01116 | P10398 | KRAS | ARAF | 0.923 | 0.875 | - |
| P01116 | P01116 | KRAS | KRAS | 1 | 0.875 | - |
| P01112 | Q9NQU5 | HRAS | PAK6 | 0.833 | 0.875 | - |
| O75914 | Q9P286 | PAK3 | PAK7 | 0.875 | 0.714 | - |
| O75914 | Q9NQU5 | PAK3 | PAK6 | 0.875 | 0.714 | - |
| P01112 | Q07890 | HRAS | SOS2 | 0.929 | 0.875 | - |
| O75914 | Q13177 | PAK3 | PAK2 | 0.875 | 0.714 | - |
| O75914 | O75914 | PAK3 | PAK3 | 0.929 | 0.714 | - |
| P01112 | P01116 | HRAS | KRAS | 1 | 0.812 | - |
| P01112 | P01112 | HRAS | HRAS | 1 | 0.9 | - |
| P01111 | Q9NQU5 | NRAS | PAK6 | 0.829 | 0.771 | - |
| P01111 | Q07890 | NRAS | SOS2 | 0.929 | 0.771 | - |
| P01111 | Q07889 | NRAS | SOS1 | 0.929 | 0.8 | - |
| P01111 | P10398 | NRAS | ARAF | 0.923 | 0.771 | - |
| P01111 | P01116 | NRAS | KRAS | 0.929 | 0.8 | - |
| P01111 | P01112 | NRAS | HRAS | 1 | 0.9 | - |
| P01111 | P01111 | NRAS | NRAS | 1 | 0.9 | - |
